# Clostridium difficile colonization in preoperative colorectal cancer patients

**DOI:** 10.18632/oncotarget.14424

**Published:** 2017-01-02

**Authors:** Yi Zheng, Yun Luo, Yinxiang Lv, Chen Huang, Qinsong Sheng, Peng Zhao, Julian Ye, Weiqin Jiang, Lulu Liu, Xiaojun Song, Zhou Tong, Wenbin Chen, Jianjiang Lin, Yi-Wei Tang, Dazhi Jin, Weijia Fang

**Affiliations:** ^1^ Cancer Biotherapy Center, First Affiliated Hospital, School of Medicine, Zhejiang University, Hangzhou, China; ^2^ Department of Microbiology, Zhejiang Provincial Center for Disease Control and Prevention, Hangzhou, China; ^3^ Xinchang People's Hospital, Shaoxing, China; ^4^ Department of Colorectal Surgery, First Affiliated Hospital, School of Medicine, Zhejiang University, Hangzhou, China; ^5^ Department of Laboratory Medicine, Memorial Sloan Kettering Cancer Center; Department of Pathology and Medicine, Weill Medical College of Cornell University, New York, NY, USA

**Keywords:** Clostridium difficile, colorectal cancer, colonization, epidemiology, transmission

## Abstract

The entire process of *Clostridium difficile* colonization to infection develops in large intestine. However, the real colonization pattern of *C. difficile* in preoperative colorectal cancer patients has not been studied. In this study, 33 *C. difficile* strains (16.1%) were isolated from stool samples of 205 preoperative colorectal cancer patients. *C. difficile* colonization rates in lymph node metastasis patients (22.3%) were significantly higher than lymph node negative patients (10.8%) (OR=2.314, 95%CI=1.023-5.235, *P* =0.025). Meanwhile, patients positive for stool occult blood had lower *C. difficile* colonization rates than negative patients (11.5% vs. 24.0%, OR=0.300, 95%CI=0.131-0.685, *P* =0.019). A total of 16 sequence types were revealed by multilocus sequence typing. Minimum spanning tree and time-space cluster analysis indicated that all *C. difficile* isolates were epidemiologically unrelated. Antibiotic susceptibility testing showed all isolates were susceptible to vancomycin and metronidazole. The results suggested that the prevalence of *C. difficile* colonization is high in preoperative colorectal cancer patients, and the colonization is not acquired in the hospital. Since lymph node metastasis colorectal cancer patients inevitably require adjuvant chemotherapy and *C. difficile* infection may halt the ongoing treatment, the call for sustained monitoring of *C. difficile* in those patients is apparently urgent.

## INTRODUCTION

*Clostridium difficile* infection (CDI) is one of the leading causes of antibiotic-associated diarrhea. World-widely, the incidence of CDI has increased significantly since ribotype 027 strains appeared at the beginning of the century. There have been a lot of outbreaks with severe cases reported in the United States and Europe. A recent study indicated that approximately 453,000 cases of CDI and 29,000 deaths associated with CDI were identified each year in the United States [1]. The risk factors of CDI include antibiotic exposure, advanced age and hospitalization, which have been reported in detail and widely accepted [2–5]. Cancer patients who were immunocompromised were reported to have a higher risk for CDI compared with non-cancer patients. It is due of antibiotic-like activity of several chemotherapy drugs and chemotherapy-induced neutropenia [6, 7].

*C. difficile* mainly colonizes the large bowel as a part of normal intestinal flora. There are toxigenic and non-toxigenic strains. Toxigenic strains release exotoxins TcdA and TcdB to result in colitis and other diseases. Prevalence of *C. difficile* colonization for ICU patients [8], cancer patients [9, 10], patients undergoing hematopoietic stem cell transplantation [11, 12], residents in long-term care facilities [13], and healthy people [14] has been previously reported. The rates of *C. difficile* colonization in adult patients are different in different regions, but significantly lower than those in children [15]. A substantial proportion of individuals in the asymptomatic population with *C. difficile* colonization have been demonstrated to serve as reservoirs for CDI [16]. Although the carriage of nontoxigenic *C. difficile* strains might prevent CDI in humans, toxigenic *C. difficile* colonization is regarded as an independent risk factor prone to developing CDI subsequently [15]. The risk factors of *C. difficile* colonization include previous hospitalization [9, 15, 17], previous exposure to antibiotics [15], the use of gastric acid-suppressing drugs [18], and host variables [8–15].

The intestinal microbiota is also believed to be directly involved in colorectal carcinogenesis [19]. However, the rate of *C. difficile* colonization in preoperative colorectal cancer (CRC) patients has not been previously reported. The main transmission pattern and specific risk factors of *C. difficile* colonization in hospitalized CRC patients remains unclear.

Here we performed a preliminary study to investigate the pattern of *C. difficile* colonization in preoperative CRC patients admitted in a tertiary teaching hospital in China. The aim of this study is to reveal the rate of *C. difficile* colonization and its correlation to clinical characteristics in preoperative CRC patients. In addition, genotypes and antibiotic resistance profiles of *C. difficile* strains in those patients were also analyzed.

## RESULTS

### Collection of *C. difficile* isolates

A total of 205 preoperative CRC patients were included in this study. Among them, Thirty-three (16.1%) were positive for *C. difficile*. Among these thirty-three *C. difficile* isolates, twenty-eight (84.8%) were positive for both *tcd*A and *tcd*B (A^+^B^+^), four (12.1%) were negative for *tcd*A and positive for *tcd*B (A^-^B^+^), and one isolate (3.0%) was non-toxigenic with neither *tcd*A nor *tcd*B (A^-^B^-^). Toxigenic isolates were dominant at a rate of 97.0%. There was only one type of *C. difficile* isolate detected in each culture-positive stool specimen.

### Clinical characteristics of CRC patients and *C. difficile* colonization

There were 134 (65.4%) male and 71 (34.6%) female patients. *C. difficile* colonization rate in male patients was 15.7%, whereas in female patients was 16.9% (*P* = 0.820). The mean age is 63.3 years old. The mean ages of the toxigenic *C. difficile* positive and *C. difficile* negative patients were 64.1 and 62.2 years old, respectively. As shown in Table 1, there is a tendency of higher *C. difficile* colonization rate in patients over 60 years old (*P* = 0.249). Among all CRC patients, 29 of the 173 (16.8%) left-sided CRC patients were positive for *C. difficile*, and 4 of 32 (12.5%) right-sided CRC patients were positive for *C. difficile* (*P* = 0.547), no colonization preference was found between different sides.

**Table 1:** Characteristics and risk factors of *C. difficile* colonization in 205 CRC patients

Characteristic	No.(%) of patients for *C. difficile* status		Results for analysis			
	*C. difficile* positive	*C. difficile* negative	Bivariant		Multivariant logistic	
	(n=33)	(n=172)	*P* value	OR	95% CI	*P* value
Age	<60 (n=74)	9 (12.2)	65 (87.8)	0.249	1.560	0.652-3.735	0.318
	≥60 (n=131)	24 (18.3)	107 (81.7)				
Site	Colon (n=91)	15 (16.5)	76 (83.5)	0.893	0.991	0.397-2.474	0.984
	Rectum (n=114)	18 (15.8)	96 (84.2)				
Morphology	Ulcerative (n=134)	25 (18.7)	109 (81.3)	0.171	0.744	0.287-1.926	0.242
	Exophytic (n=71)	8 (11.3)	63 (88.7)				
Differentiation	Poor (n=62)	8 (12.9)	54 (87.1)	0.413	1.424	0.825-6.481	0.401
	Well (n=143)	25 (17.5)	118 (82.5)				
T stage	Non-T4 (n=150)	20 (13.3)	130 (86.7)	0.075	2.301	0.963-5.500	0.061
	T4 (n=55)	13 (23.6)	42 (76.4)				
N stage	LN neg. (n=111)	12 (10.8)	99 (89.2)	0.025*	2.314	1.023-5.235	0.044*
	LN pos. (n=94)	21 (22.3)	73 (77.7)				
Albumin (g/L)	<35 (n=17)	5 (29.4)	12 (70.6)	0.119	0.315	0.073-1.357	0.121
	≥35 (n=188)	28 (14.9)	160 (85.1)				
FBG (mmol/L)	<7.0 (n=192)	32 (16.7)	160 (83.3)	0.394	0.337	0.033-3.455	0.360
	≥7.0 (n=13)	1 (7.7)	12 (92.3)				
BMI	<24 (n=114)	17 (14.9)	97 (85.1)	0.605	1.358	0.581-3.177	0.480
	≥24 (n=91)	16 (17.6)	75 (82.4)				
TG (mmol/L)	≥1.70 (n=38)	6 (15.8)	32 (84.2)	0.954	1.129	0.497-4.703	0.917
	<1.70 (n=167)	27 (16.2)	140 (83.8)				
TC (mmol/L)	<5.70 (n=190)	31 (16.3)	159 (83.7)	0.762	0.858	0.097-3.199	0.612
	≥5.70 (n=15)	2 (13.3)	13 (86.7)				
HDL (mmol/L)	< 0.80 (n=20)	2 (10.0)	18 (90.0)	0.435	1.768	0.337-9.274	0.500
	≥ 0.80 (n=185)	31 (16.8)	154 (83.2)				
Hemoglobin (g/L)	<120 (n=55)	10 (18.2)	45 (81.8)	0.623	0.911	0.319-2.602	0.861
	≥120 (n=150)	23 (15.3)	127 (84.7)				
OB	Negative (n=75)	18 (24.0)	57 (76.0)	0.019*	0.300	0.131-0.685	0.004*
	Positive (n=130)	15 (11.5)	115 (88.5)				

Abbreviations: *P <0.05; LN: Lymph nodes; FBG: Fast blood glucose; BMI: Body mass index; TG: Triglyceride; TC: Total cholesterol; HDL: High density lipoprotein

Pathological features including cancer sites of origin (colon to rectum), macroscopic morphology (exophytic to ulcerative), and differentiation (well to poor) of the CRC patients were then analyzed for correlation with *C. difficile* colonization. However, as shown in Table 1, no clear correlation was identified between the rate of colonization and these pathological features. In addition, whether the CRC patients are more prone to toxigenic or non-toxigenic *C. difficile* colonization is also inconclusive (data not shown).

Generally speaking, T4 (tumor penetrate the visceral peritoneum) and lymph node (LN) metastasis are of great importance for CRC patients as poor prognostic factors and indicators for adjuvant chemotherapy. While in terms of *C. difficile* colonization in CRC patients at different stages, a comparative analysis of rate of colonization between T4 and non-T4, LN metastasis and LN negative patients was performed. Interestingly, CRC patents with high risk factors are likely to have higher *C. difficile* colonization rate than others, as shown in Table 1. It is particularly true that patients of positive LN metastasis bear significantly higher rate of *C. difficile* colonization than those of negative LN (22.3% to 10.8%, *P* =0.025). Multivariate logistic regression analysis also showed that LN metastasis is an independent risk factor for *C. difficile* colonization in CRC patients (*P* = 0.044).

CRC frequently causes chronic bleeding, inducing anemia and malnutrition. Therefore, stool OB and blood hemoglobin of the CRC patients were used as differential tools in correlation with *C. difficile* colonization. Among all 205 CRC patients studied, OB negative patients had significantly higher *C. difficile* positive rate than OB positive patients (24.0% to 11.5%, *P* =0.019). Multivariate logistic regression analysis also revealed OB negativity as an independent predictor for *C. difficile* colonization (*P* =0.004). No relationship was found between blood hemoglobin level and *C. difficile* colonization. Nutritional indexes and metabolic profiles of CRC patients, include albumin, fast blood glucose, triglyceride, total cholesterol, high density lipoprotein, and body mass index, were collected and analyzed for possible relation to *C. difficile* colonization. However, as shown in Table 1, the results revealed that none of the indexes could be interpreted as risk factor for *C. difficile* colonization in CRC patients.

In addition, whether *C. difficile* colonization has effected the duration of hospitalization was also studied. For the 172 *C. difficile* negative patients, the average days in hospital were 15.4 ± 0.34 days (mean ± s.e.m.), while the average days in hospital were 15.0 ± 0.98 days (mean ± s.e.m.) for the *C. difficile* positive patients, the result was of no statistical difference (*P* >0.05).

### Molecular typing of *C. difficile* isolates from CRC patients

**Figure 1:**
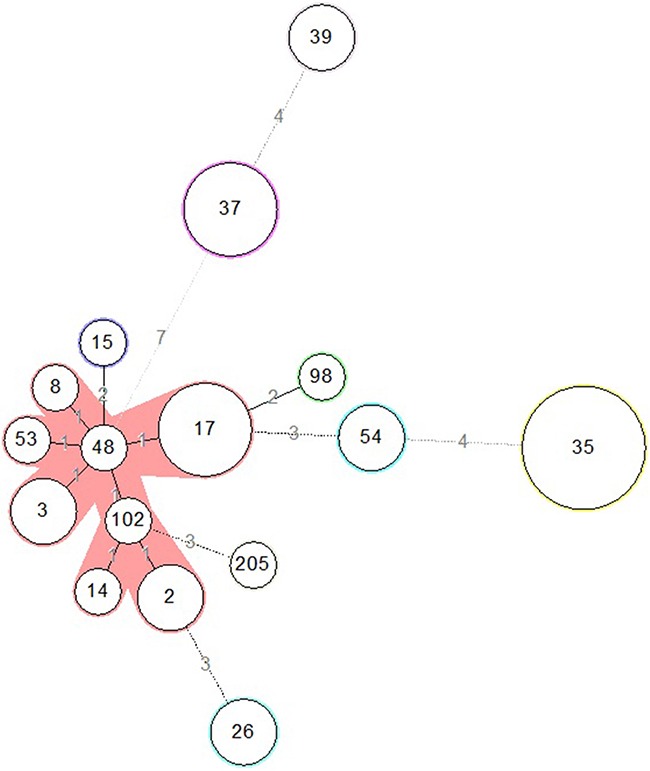
Relationships of the 33 colonizing *C. difficile* strains by minimum spanning tree based on MLST data The number in the circle indicates the ST and the size of the circle corresponds the total number of isolates belonging to that ST. The number of allelic difference between STs is indicated on the branches. Nodes were connected by a dashed line if the difference is more than two alleles.

**Figure 2:**
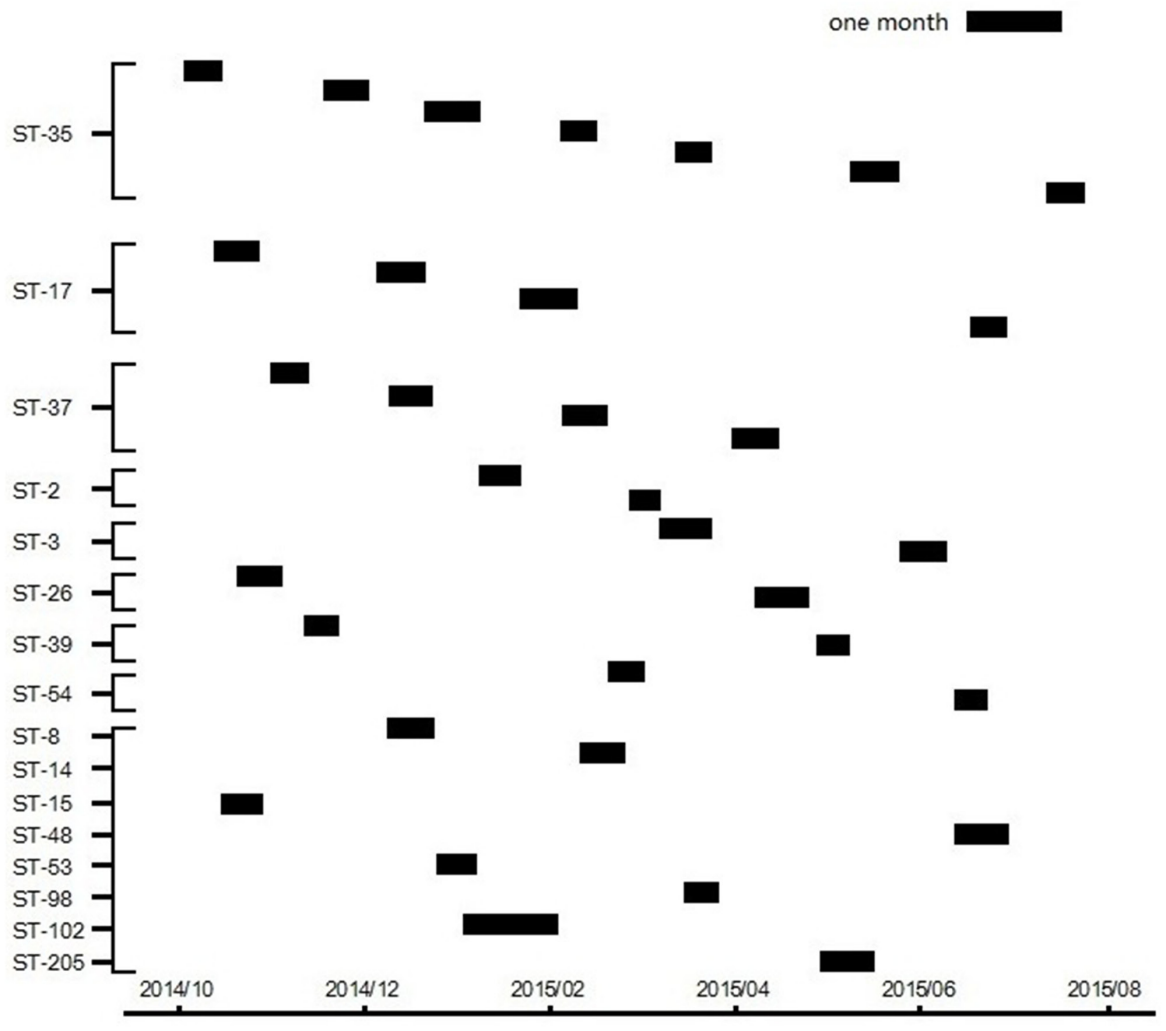
Time-space cluster map of different STs from individual CRC patients with *C. difficile* colonization Y-axis depicts unique multilocus STs. X-axis (bottom) shows the duration of the study period. Each small box represents the date of admission and the length of hospital stay of an individual *C. difficile* colonizing CRC patient.

MLST results divided the 33 *C. difficile* isolates into 16 STs, indicating a great diversity of the genotype of *C. difficile* colonization. As previously described, three toxin gene profiles were included labeled as A^+^B^+^, A^-^B^+^, and A^-^B^-^. ST35 (21.2%) was predominant which consists of 7 isolates. A minimum spanning tree was constructed (Figure 1). The eBURST analysis showed none of a single ST formed a clonal complex. Four sets of closely related STs are listed as the follows. ST37 and ST39 are in the same set, ST17, ST98, ST54 and ST35 consists of one set, ST102, ST14, ST205, ST2 and ST26 with genetic relevance belongs to one set, and the remaining STs belongs to another set. The temporospatial relationship for same STs was mapped over the study period as shown in Figure 2. No cluster of any ST was observed. There were no overlaps among these cases in each ST line in time and space. These results indicate that no cases of the 33 isolates were genetic or epidemiological related. Although all the 33 patients finally had hospital stays varies from one week to less than four weeks, they did not obviously get infected with *C. difficile* during the time of hospitalization.

### Antimicrobial resistance of *C. difficile* isolates from CRC patients

Antimicrobial resistance of the 33 *C. difficile* isolates was tested against 12 antibiotics. All the isolates were susceptible to vancomycin and metronidazole. Only one isolate was resistant to piperacillin-tazobactam. The relationship of antimicrobial resistance of the *C. difficile* isolates and clinical characteristics of CRC patients were analyzed. As shown in Table 2, patients of rectum cancer bore more fusidic acid resistant *C. difficile* isolates than those of colon cancer (OR: 7.15, 95% CI: 1.53-33.37, *P* =0.01). In addition, CRC patients over 60 years old carried more moxifloxacin susceptible isolates than patients less than 60 years old (OR: 7.00, 95%CI: 1.17-41.76, *P* =0.02). Antibiotic resistance profiles were also correlated with MLST relationships. All ST35 isolates and all but one ST37 isolates were resistant to tetracycline. All ST 37 isolates were resistant to clindamycin. All but one ST35 isolates were resistant to erythromycin (data not shown). Notably, the only one non-toxigenic isolate, ST15, was resistant to tetracycline only, but susceptible to all other antibiotics. It implies that the non-toxigenic *C. difficile* isolate had a lower level of antibiotics resistance than toxigenic *C. difficile* isolate.

**Table 2:** Antibiotic resistance profile of 33 *C. difficile* strains according to pathology and demographic characteristics

Antibiotic	No. (%) resistant						
		Cancer site			Age		
	Total	Rectum	Colon	P value	<60	≥60	P value
	n=33	n=18	n=15		n=9	n=24	
Fusidic acid	17 (51.5)	13 (72.2)	4 (26.7)	0.01*	4 (44.4)	13 (54.2)	0.62
Ciprofloxacin	26 (78.8)	14 (77.8)	12 (80.0)	0.88	9 (100.0)	17 (70.8)	0.07
Piperacillin/Tazobactam	1 (3.0)	1 (5.6)	0 (0)	NA	0 (0)	1 (4.2)	NA
Metronidazole	0 (0)	0 (0)	0 (0)	NA	0 (0)	0 (0)	NA
Rifampicin	9 (27.3)	5 (27.8)	4 (26.7)	0.94	1 (11.1)	8 (33.3)	0.20
Moxifloxacin	15 (45.5)	6 (33.3)	9 (60.0)	0.13	7 (77.8)	8 (33.3)	0.02*
Gatifloxacin	14 (42.4)	8 (44.4)	6 (40.0)	0.80	2 (22.2)	12 (50.0)	0.15
Vancomycin	0 (0)	0 (0)	0 (0)	NA	0 (0)	0 (0)	NA
Clindamycin	21 (63.6)	12 (66.7)	9 (60.0)	0.69	6 (66.7)	15 (62.5)	0.83
Levofloxacin	22 (66.7)	12 (66.7)	10 (66.7)	1.00	7 (77.8)	15 (62.5)	0.41
Tetracycline	16 (48.5)	8 (44.4)	8 (53.3)	0.53	6 (66.7)	10 (41.7)	0.20
Erythromycin	21 (63.6)	11 (61.1)	10 (66.7)	0.74	8 (88.9)	13 (54.2)	0.07

*P <0.05

## DISCUSSION

CRC is among the most commonly diagnosed malignancy all over the world [20]. Apart from the life style and pre-existed comorbidities such as ulcerative colitis and adenomas, alteration in gut microbiota is regarded as an important driving factor during the carcinogenesis of CRC. According to previous reports, *F. nucleatum*, enterotoxigenic *B. fragilis* and adherent-invasive *E. coli* are all found to promote the adenoma-carcinoma sequence [21–24].

So far the exact colonization pattern of *C. difficile* in CRC patients has not been determined yet. In a prospective study, 19% of patients were colonized with toxigenic *C. difficile* on admission to oncology [25]. The rate of *C. difficile* colonization in admitted children in hematologic ward was reported to be 25.6%, with a 92.6% of toxigenic strains [10]. Our previous study revealed a 20.5% of toxigenic *C. difficile* colonization in cancer patients [9]. No symptomatic CDI patients were revealed in our study although the prevalence of *C. difficile* colonization in preoperative CRC patients was high in China from our clinical experience. We speculated that antiemetic pharmaceuticals might inhibit patients’ diarrhea symptoms to some extent in the course of ongoing treatment. Furthermore, CDI clinical severity is generally mild to moderate in Chinese patients in our other studies [26]. However, the data remain scanty. Even though CDI has been prevalent in 15% of cancer patients receiving chemotherapy [27], the relationship between *C. difficile* colonization and CDI in cancer patients remains unclear. Further investigations are needed to clarify the risk factors triggering the transformation from *C. difficile* colonization to CDI in cancer patients.

Stage T4 and LN metastasis are both high risk factors for recurrence and indications for adjuvant chemotherapy in CRC patients. Our results indicated that CRC patients with more advanced disease (T4 or LN metastasis) who definitely need adjuvant chemotherapy after surgery tend to have higher rate of *C. difficile* colonization. It is accepted that the colonization of *C. difficile* in large intestine is prevented by the barrier of the gut microbiota. Weakening of this resistance by cancer is the major risky condition leading to infection. Differences in colon microbiota between individuals with a normal colonoscopy and CRC have been reported [28, 29]. In addition, relatively longer disease course and more aggressive treatment in patients with stage T4 and LN metastasis could compromise the protection of gut microbiota and facilitate *C. difficile* colonization. From the pathological perspective, changes in the composition of the gut microbiota may lead to the instability of homeostasis, resulting inflammation, dysplasia, and carcinogenesis [30–32]. Presence and overgrown of *C. difficile* in CRC patients, especially during the adjuvant chemotherapy, might develop CDI and result in severe diarrhea, which in turn halts the ongoing chemotherapy [33]. Therefore, *C. difficile* colonization should be examined for all preoperativeCRC patients as a part of risk stratification for further cancer therapy.

There were no clear correlation between the rate of *C. difficile* colonization and the sites of origin, histological morphology, and metabolic factors of the cancer. Although a previous study demonstrated a predictable role of albumin in CDI [34], none of the metabolic factors described here seems to be capable to predict *C. difficile* colonization in CRC patients. However, we notably found OB negative patients had a higher rate of *C. difficile* colonization. Previous studies also disclosed that there are extremely few CDI patients with positive fecal OB [35]. The possible reason underlying this finding is that bleeding from the cancer lesions might change microbiota and microenvironment, leading to the alteration of *C. difficile* colonization. Moreover, blood components might also hinder *C. difficile* colonization or CDI. More studies are needed in order to clarify the relationship between OB and *C. difficile* colonization

There was a specific correlation between antibiotic resistance and clinical characteristics, indicating that the individualized therapeutic scheme should be considered according to clinical characteristics of the patients. All the isolates in this study were susceptible to metronidazole and vancomycin with low minimal inhibitory concentration. Besides, the resistant rate to piperacillin-tazobactam is relatively low, which suggests it can serve as an alternative option for treatment of CDI in CRC patients. The non-toxigenic isolate was resistant only to tetracycline, indicating a significant narrower antibiotic resistance in the non-toxigenic isolates than that in the toxigenic isolates.

Compared with hospital-acquired infection, morbidity and mortality associated with community-acquired *C. difficile* colonization are lower [36]. All the cases in this study were genetically and epidemiologically unrelated to one another based on MLST analysis and epidemiological data. We also investigated the molecular typing of *C. difficile* from same patients 48 hours after admission. The results showed the molecular spectrum of *C. difficile* isolates was still wide (data not shown), suggesting these patients were community-acquired according to the 2010 SHEA/IDSA guideline [7]. The findings can be possibly used as reliable data for further investigation of the *C. difficile* transmission pattern in China.

In conclusion, this is a preliminary study in determining prevalence and revealing risk factors of *C. difficile* colonization in CRC patients in China. CRC patients harboring high risk factors such as T4 or LN metastasis tended to have higher *C. difficile* colonization rate. Therefore, peri-operative screening and monitoring for *C. difficile* is of great importance in these patients in order to avoid discontinuation of chemotherapy due to severe diarrhea and postoperative complications. Besides, OB negativity increases risk of *C. difficile* colonization in CRC patient. As high as 97.0% of colonized *C. difficile* strains were toxigenic with multiple antibiotics resistance. *C. difficile* colonization may not be mainly acquired in hospitals due to the absence of epidemiologic relatedness in preoperative CRC patients. A larger-population-based study and mechanism-specific research is needed in the future in order to clarify the exact role of *C. difficile* colonization in CRC patients.

## MATERIALS AND METHODS

### Design of the study

Stool samples were collected from consecutive preoperative CRC patients who were scheduled to accept radical cancer resection in Department of Colorectal Surgery, the First Affiliated Hospital of Zhejiang University. The study protocol was approved by the Institutional Ethics Committees of the hospital and the study was performed in accordance with the guidelines recommended by National Institute of Health involving human subjects and animal care and 1975 Declaration of Helsinki. All patients provided written consent. Stool samples within 24 hours of admission were collected from each patient, and delivered to Department of Microbiology, Zhejiang Provincial Center for Disease Control and Prevention for culture, identification of toxin gene and antibiotic susceptibility testing.

### Patients

The study was performed between October, 2014 and August, 2015. All CRC patients were to undergo surgical resection after stool sample collection, with no preoperative chemotherapy received. No notable long-term diarrhea was reported by the patients. No antibiotics were used prior to the stool sample collection. Re-admitted patients were excluded. Demographic information and pathological findings collected from the patients include age, gender, body mass index, cancer stages (T stage: Depth of primary cancer infiltration; N stage: Regional lymph nodes metastasis), cancer differentiation, and sites of origin. Laboratory results such as blood hemoglobin level, stool occult blood (OB), fasting blood glucose, serum albumin, triglyceride, total cholesterol, and high density lipoprotein were also included.

### Stool culture and *C. difficile* isolation

*C. difficile* isolation from the stool collected within 24 hours of admission was processed as the follows. Specimens were firstly treated with alcohol. The mixture was then inoculated into cefoxitin-cycloserine fructose agar (CCFA) plates (UK Bio, Hangzhou, China). After incubation for 48 hours at 37°C in a GENbag anaerobic chamber (BioMérieux, Marcy l’Etoile, France), up to ten suspected colonies for each stool specimen were analyzed and confirmed to be *C. difficile* as described by Loo *et al* [37]. The *C. difficile* isolates were stored within the blood broth in a preservation kit (UKbio, Hangzhou) at -80°C.

### Identification of toxin gene and bioinformatics

All bacterial colonies for each stool specimen were identified. Bacterial genomic DNAs were extracted using DNeasy Blood & Tissue kit (QIAGEN, Inc., Valencia, CA) according to the manufacturer's instructions. A multiplex real time PCR assay provided by the UK Bio company in Hangzhou was used for detection of the two toxin genes (*tcd*A and *tcd*B) in order to determine between non-toxigenic and toxigenic isolates. Seven housekeeping genes (*adk*, *atp*A, *dxr*, *gly*A, *rec*A, *sod*A, and *tpi*) selected based on a previous study by Griffiths *et al*. were used for multilocus sequence typing (MLST) [38]. After the standard procedure of PCR, the amplified products were sequenced. Data was uploaded into a public *C. difficile* MLST database, accessible at http://pubmlst.org/cdifficile, for determining sequence types (ST). The eBURST was used to identify clonal complexes, each of those are defined by one of the seven typed genes, which bear different features from one another. Minimum spanning tree that reveals the allelic difference between isolates of the seven housekeeping genes was constructed by application of Bionumerics software (Applied Math). The Simpson's index of diversity (D value) was calculated with an in-house program, MLEECOMP package.

### Antibiotic susceptibility testing

*In vitro* testing for susceptibilities of antibiotics including fusidic acid, ciprofloxacin, levofloxacin, moxifloxacin, gatifloxacin, piperacillin-tazobactam, rifampicin, clindamycin, erythromycin, tetracycline, metronidazole and vancomycin was carried out using an E-test assay according to the supplement from Clinical and Laboratory Standards Institute (CLSI)[39]. Six E-test strips (BioMérieux, Marcy l’Etoile, France) were tested in one plate with brucella broth agar. Results were analyzed and categorized as susceptible, intermediate or resistant according to the CLSI breakpoints and other previous studies [39–41].

### Statistical analysis

Statistical analysis was performed by applying SPSS 20.0 (Chicago, IL, USA). Categorized data were analyzed with the chi-squared test. The F-test and T-test were conducted for hetero or equal variance analysis. Significance of characteristics in individual patients was determined by multivariate logistic regression analysis. Statistical significance for all tests was defined as *P* < 0.05.
